# Health Benefits and Pharmacological Aspects of Chrysoeriol

**DOI:** 10.3390/ph15080973

**Published:** 2022-08-07

**Authors:** Sara Aboulaghras, Nargis Sahib, Saad Bakrim, Taoufiq Benali, Saoulajan Charfi, Fatima-Ezzahrae Guaouguaou, Nasreddine El Omari, Monica Gallo, Domenico Montesano, Gokhan Zengin, Khalid Taghzouti, Abdelhakim Bouyahya

**Affiliations:** 1 Physiology and Physiopathology Team, Faculty of Sciences, Genomic of Human Pathologies Research, Mohammed V University in Rabat, Rabat 10100, Morocco; 2 Laboratoire d’Amélioration des Productions Agricoles, Biotechnologie et Environnement (LAPABE), Faculté des Sciences, Mohammed Premier University, Oujda 60000, Morocco; 3 Geo-Bio-Environment Engineering and Innovation Laboratory, Molecular Engineering, Biotechnologies and Innovation Team, Polydisciplinary Faculty of Taroudant, Ibn Zohr University, Agadir 80000, Morocco; 4 Environment and Health Team, Polydisciplinary Faculty of Safi, Cadi Ayyad University, Marrakesh-Safi 46030, Morocco; 5 Laboratory of Biotechnology and Applied Microbiology, Department of Biology, Faculty of Sciences, Abdelmalek Essaadi University, Tetouan 93030, Morocco; 6 LPCMIO, Materials Science Center (MSC), Ecole Normale Supérieure de Rabat, Mohammed V University in Rabat, Rabat 10100, Morocco; 7 Laboratory of Histology, Embryology, and Cytogenetic, Faculty of Medicine and Pharmacy, Mohammed V University in Rabat, Rabat 10100, Morocco; 8 Department of Molecular Medicine and Medical Biotechnology, University of Naples Federico II, Via Pansini 5, 80131 Naples, Italy; 9 Department of Pharmacy, University of Naples Federico II, Via D. Montesano 49, 80131 Naples, Italy; 10 Department of Biology, Science Faculty, Selcuk University, Konya 42250, Turkey; 11 Laboratory of Human Pathologies Biology, Department of Biology, Faculty of Sciences, Mohammed V University in Rabat, Rabat 10100, Morocco

**Keywords:** chrysoeriol, biological activities, pharmacokinetic, pharmacodynamic

## Abstract

A flavone, chrysoeriol is synthetized in several plant species. It comes from several natural sources, especially medicinal plants. The identification and isolation of this compound has been carried out and verified by several research teams using different spectral methods. It seems that the concentration of this molecule is variable and fluctuating depending on the source, the part extracted, the region, and the methods of extraction and characterization. The aim of this paper is to highlight the in vitro and in vivo pharmacological properties of chrysoeriol and to provide insight into its pharmacokinetics. Anticancer, anti-inflammatory, antibacterial, antifungal, anti-osteoporosis, anti-insecticide, and neuroprotective actions have been shown in a number of studies on this chemical. Different mechanisms in theses pharmacological effects include subcellular, cellular, and molecular targets. In vivo pharmacokinetic analysis has proved the good stability of this molecule, showing its promising potential to prevent or treat diseases including cancer, diabetes, inflammation, osteoporosis, Parkinson’s disease, and cardiovascular diseases.

## 1. Introduction

Chrysoeriol is a 30-O-methoxy flavone, which means that it is a chemically derived product of luteolin, which is a substance that is part of the flavone family of chemicals. It may be produced in a variety of plant species, such as *Coronopus didymus* (L.) Sm. [1,2], *Capsicum* sp. [3,4], *Eurya cilliata* Merr. [5,6], *Eremosparton songoricum* (Litv.) Vass. [7,8], and *Medicago sativa* L. [9]. Several studies have showed that chrysoeriol has a wide range of pharmacological activities. Indeed, this molecule has exhibited significant anticancer activity against different tumor cell lines, including cervical cancer [5,10], human lung cancer [10,11], and colon cancer cell lines [5,12]. In addition, the anticancer effect of chrysoeriol has been reported in vivo [11,13] and through in silico molecular docking against several cancer receptors [14]. On the other hand, chrysoeriol has showed an anti-inflammatory effect in several in vitro studies [15,16,17,18,19,20]. In animal models, it has ameliorated acute skin inflammation [21] and exhibited reno-protection against acute kidney injury [22]. Additionally, chrysoeriol has proved to be a promising antidiabetic compound in vitro [23,24,25,26] and in animal models [27,28,29,30,31,32]. It has also exhibited anti-hyperlipidemic activity in diabetic rats [33]. Furthermore, chrysoeriol has had antibacterial [3,34,35], antifungal [4], and anti-osteoporosis activities [6]. Moreover, chrysoeriol has exhibited neuroprotective action in rats [36] and in the treatment of Parkinson’s disease [37]. It has also exhibited interesting potential in the prevention and treatment of cardiovascular diseases [7,38,39]. On the other hand, chrysoeriol has exhibited anti-insecticidal activity against *Acyrthosiphon pisum*, *Rhizobium meliloti* [9], and *Spodoptera litura* [40]. Its use as antioxidant has also been reported [2,3,4,5,8,30,41,42,43]. In contrast, chrysoeriol has good properties of stability and pharmacokinetic behavior [44], allowing its pharmacological use. As a result, the objective of this study was to provide a critical assessment of chrysoeriol’s pharmacological characteristics and in vivo pharmacokinetics.

## 2. Sources of Chrysoeriol

Polyphenols are commonly subdivided into tannins, lignins, flavonoids, and anthocyanins, which all derive from the assembly of phenolic units. In particular, flavonoids are known for their antioxidant, antibacterial, antiviral, anti-inflammatory, antiproliferative, and enzyme system regulatory properties. These functions are very frequently connected to their antioxidant activity and in particular their capacity to trap free radicals, chelate metal ions, or block the enzymes responsible for the generation of radicals.

**Figure 1:**
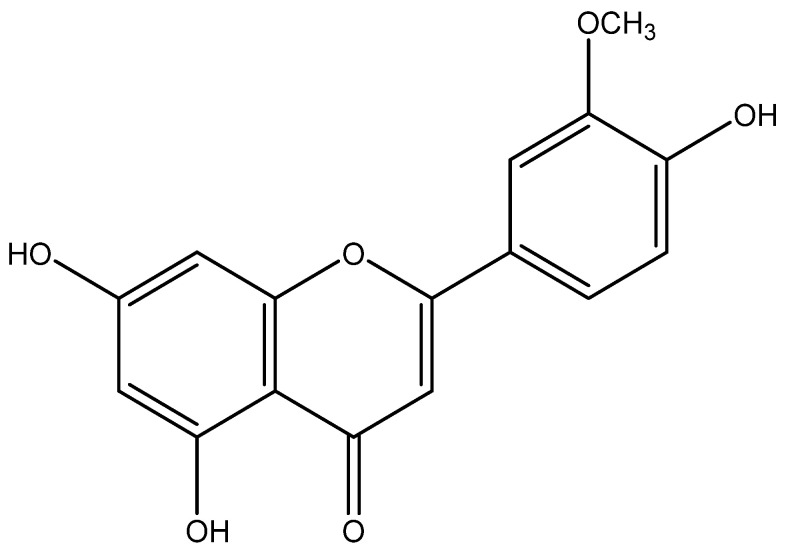
Chemical structure of chrysoeriol.

In this review, we are interested in the natural sources of chrysoeriol (Figure 1), as well as the analytical methods used for their characterization and identification. Various works have shown the presence of chrysoeriol and their derivatives in several plants in different countries of the world (Table 1). Identification of the chemical composition of *Melientha suavis* Pierre extracts showed that *M. suavis* is rich in chrysoeriol [40]. Physical and spectroscopic characteristics were checked against those published by Bashyal et al. to confirm the purification of this chemical [34]. Flavonoids, steroids, and tannins were found in the ethyl acetate fraction of *Olea europaea* L. leaves after a rigorous study. Among the isolated flavonoids, they found chrysoeriol [45]. The latter was identified using proton (^1^H-NMR) and carbon (^13^C-NMR) nuclear magnetic spectroscopy.

Kim and Jin [4] showed that the flavonoids in the species *Capsicum chinense* Jacq. are derivatives of three basic structures: chrysoeriol, luteolin-7-*O*-glucopyranoside, and isorhamnetin-7-*O*-glucopyranoside. Additionally, chrysoeriol and its derivatives were discovered in a comprehensive study of six aromatic natural compounds extracted and described from a methanolic extract of *Narthecium ossifragum* (L.) Huds. [46].

However, *Glandularia selloi* (Spreng.) Tronc. aerial parts methanolic extract yielded two novel chrysoeriol compounds, selloside A and B. Khallouki et al. [48] were able to identify ferulic acid, rutin, luteolin, isorhamnetin, and chrysoeriol in Moroccan Medjool date. Still in Africa but this time in Cameroon, chrysoeriol, isorhamnetin, and luteolin are the flavonoids that have been isolated from the aerial portions of the *Graptophyllum grandulosum* Turill plant [49].

Chrysoeriol has already been characterized in the genus *Cardiospermum* and more particularly in *Cardiospermum halicacabum* L. [33]. In diabetic rats, oral treatment of the discovered chrysoeriol resulted in a considerable reduction in blood glucose levels and an increase in insulin levels as compared to diabetic control rats. GC–MS (gas chromatography–mass spectrometry) analysis of *Arum palaestinum* Boiss. extracts further demonstrated the isolation and characterization of four compounds: luteolin, chrysoeriol, isoorientin, and isovitexin. Chrysoeriol was isolated for the first time in this plant [50]. This identification was successful thanks to the conditions followed. These conditions were quoted in the publication of Farid et al. [50] as follows: GC–MS analysis was performed on a Thermo Scientific TRACE GC ultra-gas chromatograph directly coupled to an ISQ single quadrupole mass spectrometer by using non-polar 5% phenyl methylpolysiloxane capillary column (30.00 m × 0.25 mm × 0.25 mm) type TG-5MS under the following conditions: oven temperature program from 60 °C (3 min) to 280 °C at 5 °C/min, and then isothermal at 280 °C for 5 min. The carrier gas was helium at a flow rate of 1 mL/min. The volume of injected sample was 1 mL of sample in diethyl ether using the splitless injection technique, ionization energy 70 eV, in the electronic ionization mode [50].

Benyahia et al. [51] reported that phytochemical analysis of *Artemisia arborescens* L. native to Algeria succeeded in the isolation of 15 flavonoids, including eight flavones and six flavonols. Among the identified flavonoids, they found artemetine, apigenin, chrysoeriol, and its derivatives. The chemical composition of the seeds, barks, and whole fruits of *Capsicum frutescens* L. includes capsaicin, dihydrocapsaicin, and chrysoeriol [3].

The work of Demirtas et al. [41] has shown the existence of a wide variety of chemicals in the extracts of *Allium vineale* L. In addition to that, they were successful in isolating a number of flavonoids, such as chrysoeriol and isorhamnetin. On the other hand, and specifically from Eastern Europe (Poland), the phytochemical analysis by mass spectroscopy of *M. sativa* extracts showed the richness of the aerial parts of this plant by luteolin, apigenin, tricin, and chrysoeriol [52,53]. A comprehensive analysis of the genus *Saussurea* (from Russia and Mongolia), namely *Saussurea alpina* L., *Saussurea daurica* L., *Saussurea laciniata* L., *Saussurea pricei* L., *Saussurea pseudo-alpina* L., *Saussurea salicifolia* L., and *Saussurea salsa* L. by Iwashina et al. [54] resulted in identification of thirteen flavonoids belonging to flavones and flavonols. Among the isolated flavonoids are apigenin, luteolin, chrysoeriol, and their derivatives [54]. On the other side of Asia, and more specifically in Japan, fourteen flavonoids have been characterized in the methanol extract of *Myoporum bontiodes* by Iwashina and Kokubugata [55]. Examples of these flavonoids are apigenin 7-*O*-glucuronide, luteolin 7-*O*-glucoside, luteolin 7-*O*-glucuronide, chrysoeriol 7-*O*-glucoside, chrysoeriol 7-*O*-glucuronide, selagine 7-*O*-glucoside, selagine 7-*O*-glucuronide, tricine 7-*O*-glucuronide, quercetin 3-methyl ether, chrysoeriol, isokaempferide, apigenin, and luteolin [55].

The phytochemical analysis by the HPLC technique of *Eurya ciliata Merr.* extracts carried out by Kim et al. [5] revealed the presence of chrysoeriol, and the latter showed its effectiveness in protecting osteoblasts from oxidative-stress-induced toxicity.

The work of Tai et al. [38] on the same plant (*E. ciliata*) from Vietnam also isolated chrysoeriol as the main constituent of this plant. This identification has been proven by ^13^C NMR spectrum analysis that takes into account heteronuclear single quantum coherence, heteronuclear multiple bond correlation, and Overhauser nuclear amplification [38]. Using bombardment mass spectrometry, infrared spectrometry, H-NMR, and ^13^C-NMR spectrometry, Liu et al. [38] identified chrysoeriol as the main compound of *E. Songoricum* extracts. They also showed the purification of this compound, which was greater than 99%. Seven free radical scavenging phenolic compounds, comprising five flavonoids, rutin, chrysoeriol 7-O-rutinoside, kaempferol 3-O-glucoside, chrysoeriol 7-O-glucoside, and naringenin, were identified by HPLC from the aerial portions of the Iranian medicinal plant *Phlomis caucasica* Rech. [56]. To carry out this analysis, two preparations were the subject of this study. One is a preparation preparative reversed-phase high-performance liquid chromatography (HPLC) analysis (Shim Pak Prep-ODS column 10 lm, 250 mm 9 21.2 mm; mobile phase: 0–50 min gradient 30–50% MeOH in water; 50–62 min, isocratic MeOH 50% in water; flow-rate 20 mL/min; detection at 248 nm) and the second preparative reversed-phase HPLC analysis (Shim Pak Prep-ODS column 10 µL 250 mm × 21.2 mm; mobile phase: 0–50 min gradient 20–60% ACN in water; 50–62 min, isocratic ACN 60% in water; flow-rate: 20 mL/min, detection at 248 nm) [56].

Based on the comparison of NMR spectral data of methanol and aqueous extracts of *Zea mays* L. with published spectral data, Suzuki et al. [57] were able to identify chrysoeriol, vanillin, and vanillic acid in the methanol extract of *Z. mays*. The methanol extract was separated by chromatography on Diaion HP-20 using steps of 50% aqueous MeOH, MeOH, and acetone [57].

Based on UV and MS data, chrysoeriol was identified in *Phlomis fruticosa* L. extracts from the Balkans [58]. New compounds named chrysoeriol quercetin, kaempferol, and their derivatives were identified for the first time by EIMS, ^1^H-NMR, ^13^C-NMR, HMQC, HMBC, and UV spectroscopy in the extracts of *Schouwia thebica* Webb and *Alhagi maurorum* Medik. preventing from Egypt [59].

Moreover, in Africa and more specifically in South Africa, the phytochemical analysis of *Aspalatus linearis* (Burm.f.) R.Dahlgr. extracts proved to be rich in chrysoeriol and orientin [39]. Three novel flavonoids have been discovered in *Sideritis ozturkii’s* aerial parts. The primary chemical isolated is chrysoeriol [62]. The structures have been elucidated mainly by spectroscopic methods. To study the antidiabetic activity, Han et al. [2] chose to work on *Salix matsudana*. The ethanol extract of *S. matsudana* leaves was rich in apigenin, luteolin, chrysoeriol, and their derivatives. These compounds showed strong α-amylase inhibitory activity. It should be noted that this identification was carried out through proton and carbon nuclear magnetic resonance (1H and 13C-NMR), and spectra were measured at 300 MHz and 75 Hz, respectively, on a Brucker AC-300 (Berlin, Germany).

The work of Mishra et al. [2] and Prabhakar et al. [1] on *C. didymus* extracts from India showed that chrysoeriol and its derivatives are natural flavonoids extracted from this tropical plant. They have been isolated and characterized based on chemical and spectral studies. Stochmal et al. [63] also studied *M. sativa* extracts, and they isolated and identified ten flavones from the aerial parts of *M. sativa*. These included tricine, methyltricetin, chrysoeriol, and their derivatives. Finally, two new flavonoids, luteolin and chrysoeriol, were isolated from the aerial parts of *Phlomis nissolii* L. [64].

## 3. Biological Properties

**Figure 2:**
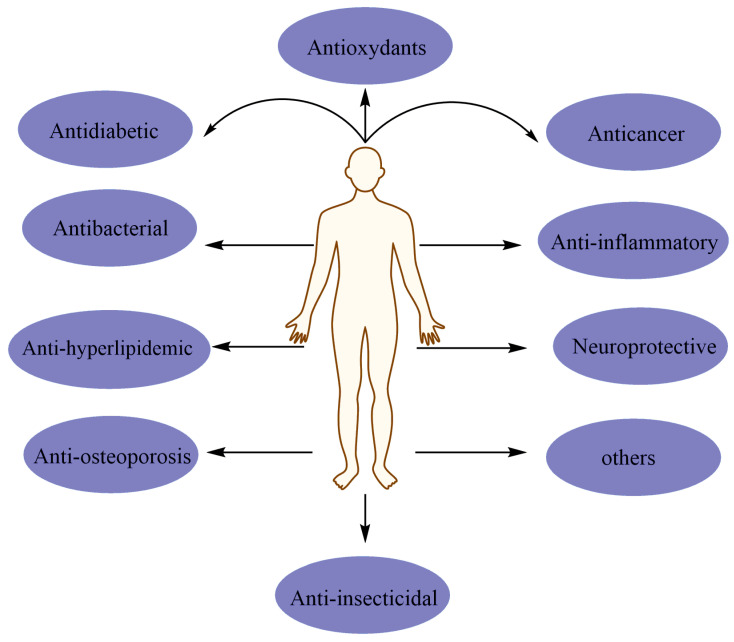
Main biological activities of chrysoeriol on human pathologies.

Different investigations have showed that chrysoeriol exhibits several pharmacological activities, and these effects involve a versatility of cellular and molecular actions (Figure 2). In the following sections, we discuss and highlight all the bioactivities of chrysoeriol with an emphasis on the main actions involved.

### 3.1. Anticancer Properties

Chrysoeriol has been shown to have strong anticancer effect on a variety of tumor cell lines, and it might be considered a unique approach to cancer treatment (Table 2). Wei and colleagues [11] assessed the anticancer property of chrysoeriol against human lung cancer cells A549. The results revealed that this molecule exerted a significant antiproliferative activity against the studied cell line (IC_50_ = 15 μM) with a lower level of cytotoxicity towards normal MRC-5 cells (IC_50_ = 93 µM). This effect was mediated through the induction of autophagy resulting from autophagosome production, an increase of Beclin-1 and LC3-II, as well as suppression of p62 expression in A549 cells treated with chrysoeriol. In addition, this compound was proven to cause a notable increase in the proportion of A549 lung cancer cells (from 0.79% to 78.54%) in the sub-G_1_/G_0_ phase of the cell cycle. Moreover, chrysoeriol can suppress cell migration and cell invasion in the studied cells dose-dependently. Furthermore, the analysis of the cytotoxic mechanism of chrysoeriol on A549 cells exhibited a potent inactivation of the m-TOR/PI3K/AKT signaling pathway. The action of chrysoeriol was also investigated in vivo using xenograft mouse models, indicating that chrysoeriol was able to repress the growth of xenografted tumors at the dosage of 50 mg/kg.

Based on the identification of levels of reactive oxygen species (ROS) and mitochondrial dysfunction, which are involved in the tumoral process, Kim et al. [5] isolated chrysoeriol from *Brucea javanica* in order to investigate its chemopreventive effect against HT-29 colon cancer, HeLa cervical cancer, and HL-60 leukemia cells using the NF-κB assay, ROS assay, MTP assay, NF-κB, and nuclear factor activated T-cell (NFAT-1) assay. They found that chrysoeriol showed high selective cytotoxicity against the HL-60 cell line but also exhibited a potent capacity to up-regulate NFAT transcriptional pathways by amplifying intracellular ROS to higher levels in the presence of H_2_O_2_. This flavone could be a great candidate to prevent chronic lymphocytic leukemia.

Zeng et al. [10] investigated the regioselective glucuronidation of diosmetin and chrysoeriol in HeLa cells overexpressing UGT1A9. The findings showed that in HeLa-UGT1A9, Ko143, a chemical inhibitor of breast cancer resistance protein (BCRP), significantly suppresses the efflux of diosmetin and chrysoeriol glucuronides and enhances their intracellular glucuronides in a dose-dependent manner, which increases cytotoxicity in A549 and HepG2 tumor cell lines. BCRP may be important for chrysoeriol glucuronides.

In an effort to understand the anti-proliferative property of *Taraxacum coreanum*, Yamabe et al. [65] examined the bioactive compounds derived from this medicinal plant, such as chrysoeriol, for their effects on human stomach cancer AGS cells. *T. coreanum* extract (50 and 100 mg/mL) and luteolin (10 and 50 micrograms/mL) were shown to result in the activation of caspase-3 and caspase-8, as well as the cleavage of poly (ADP-ribose) polymerase, lasting 24 h after administration (PARP). Moreover, chrysoeriol isolated from the CHCl_3_ fraction showed moderate cytotoxicity against AGS cells, whereas luteolin isolated from the ethanolic fraction exhibited the highest anticancer effect. Given that the anti-apoptotic protein BCL-2 is overexpressed in SW1990 pancreatic cancers, Zhang et al. [66] reported that 8-Chrysoeriol exhibited a stronger pro-apoptotic activity against SW1990 pancreatic cancer cells in vitro through targeting to BCL-2 (IC_50_ = 56.35 ± 6.96 μM). Using structure-based virtual ligand screening, this natural compound was identified as a BH3 mimetics and showed a higher affinity to BCL-2 (Kd value of 36.1 ± 3.64 μM) revealed by microscale thermophoresis technique (MST). 8-Chrysoeriol could be a promising medicinal agent in the treatment of pancreatic cancers.

**Figure 3:**
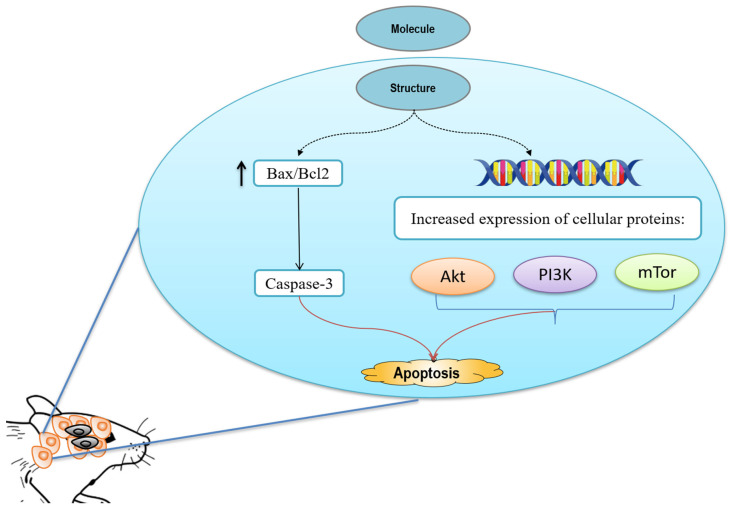
Anticancer activity of chrysoeriol against rat C6 glioma cells.

Recently, Wongkularb and collaborators [13] assessed in vivo the antineoplastic effects of this dietary methoxyflavonoid and highlighted its molecular mechanisms on rat C6 glioma cells using different methods. The results demonstrated that chrysoeriol has a potent ability to induce apoptosis (Figure 3) and notably reduce cell viability, revealed by using MTT assay. It also enhanced significantly the Bax/Bcl-2 ratio and cleaved the caspase-3/caspase-3 ratio, as well as down-regulated the phosphorylation of PI3K, Akt, and mTOR expression.

In another study, Carvalho et al. [12] investigated the antiproliferative effects of natural drugs including chrysoeriol C-glycosides extracted from the leaves and fruits of *Cydonia oblonga* Miller against A-498 and 769-P renal and Caco-2 colon cancer cells using MTT bioassay. For the fruits, the seed extracts were not effective against the growth of colon cancer cells, while the highest dose of the extract (500 μg/mL) appeared to be beneficial against A-498 and 769-P cells, with a percentage of cell growth inhibition of 91 and 84%, respectively. Indeed, the suppressive activity of 769-P and A-498 renal cells observed for the seed extract can be related to its specific phenolic composition, namely to the content of luteolin, apigenin, and chrysoeriol C-glycosides independently of the concentration of the extract, with an IC_50_ value for seed extract approximatively between 250 and 500 μg/mL. On the other hand, the leaf extract showed beneficial effects against Caco-2 colon cancer cells (IC_50_ = 239.7 ± 43.2 μg/mL), which inhibited cell growth in a concentration-dependent manner. In the same context, the objective of the study carried out by Manurung et al. [14] was to determine the anticancer effects of flavonoid agents isolated from *Plectranthus amboinicus* (Lour.) Spreng by using in silico molecular docking against several cancer receptors. The results showed that chrysoeriol, as well as other tested flavonoid products, possesses anticancer potential in the cyclooxygenase-2 and phosphoenolpyruvate carboxykinase receptors, as well as showed anticancer activity at the P-Glycoprotein-1, cyclin-dependent kinase-2, and phosphoinositide-3-kinase receptors.

The abnormal synthesis and excessive buildup of melanin may lead to skin cancer, which is why chrysoeriol was shown to protect B16F10 cells with significant efficacy against melanogenesis. According to the research, chrysoeriol significantly increased melanogenic enzyme expression, including that of the transcription factors TRP-1, TRP-2, and tyrosinase (TRY), as well as that of microphthalmia-associated transcription factor (MICF) (MITF). It also induced activation of MITF transcription in the process of melanogenesis by increasing the phosphorylation of p38 mitogen-activated protein kinase (MAPK), (GSK)-3β, β-catenin, and PKA and inhibited the production of β-catenin. This natural substance may also block the phosphorylation of ERK and protein kinase B, two more signal transduction pathways (AKT) [67].

Concerning breast cancer, using MCF7 breast cancer cells, Min et al. [68] in their research assessed the possible action of bioactive flavone, which could inhibit the expression of CYP19 through the use of different methods, including quantitative real-time PCR, immunoblot analysis, and reverse transcription PCR. They found that chrysoeriol exhibited a significant inhibitory effect on tumor necrosis factor-alpha (TNF-α)-induced EGR-1 expression using the EGR1 promoter reporter activity assay and also exerted a significant inhibitory effect on TNF-α-induced CYP19 expression via suppression of extracellular signal-regulated kinase 1/2 (ERK1/2)-mediated EGR-1 expression.

In a current survey, it was found that both SbPFOMT 2 and 5, highly expressed in the roots of *Scutellaria baicalensis* Georgi and belonging to type II of O-methyltransferase (OMTs), could methylate the 3′-hydroxyl group of luteolin to produce chrysoeriol. These enzymes have proven their critical implication in the biosynthesis of anticancer methoxylated 4′-deoxyflavones such as chrysoeriol, tenaxin I, and skullcapflavone I, which have a potent capacity to induce apoptosis in SMMC-7721 liver cancer cells, HUVEC healthy cells, and A549 lung cancer cells [70].

In order to identify bioactive drugs with anticancer properties isolated from the full plant of *Hypericum elodeoides*, Qiu et al. [71] showed that chrysoeriol (50 μmol/L) could help to suppress the 9-cis-RA-induced retinoid X receptor-α (RXRα) transcription. Moreover, chrysoeriol (12.5–50 μmol/L) showed concentration-dependent inhibitory effects. Indeed, RXRα has been shown to occupy a pivotal role in a wide range of cellular processes, and RXRα ligands have potential as novel therapeutic candidates, especially in cancer [71].

### 3.2. Anti-Inflammatory Effects

The adaptive response of inflammation is triggered by a variety of insults to tissues, including infections, endotoxins, chemicals, and other agents. It is to one’s benefit to have their inflammatory responses regulated or controlled, since this increases their degree of protection against potentially harmful stimuli [72]. Chrysoeriol has proven to possess anti-inflammatory effects in some research studies (Table 3). Indeed, Wu and colleagues have shown that chrysoeriol has an anti-inflammatory property resulting in reduced levels of inflammatory mediators and proteins, including iNOS, COX-2, phospho-p65 (Ser536), and phospho-STAT3 (Tyr705) as well as diminished generation of pro-inflammatory cytokines such as IL-1β, IL-6, and TNF-α in the mouse model. This flavonoid also has the ability to reduce the levels of prostaglandin E_2_ (PGE_2_) and NO and suppress the phosphorylation of NF-κB, Janus kinase 2 (Tyr1007/1008), and STAT3 but also down-regulate mRNA levels of pro-inflammatory cytokines IL-6, TNF-α, and IL-1β in TPA-induced inflammatory mouse ears. These findings suggested that chrysoeriol exhibited a potent inflammatory effect by inhibiting STAT3 and NF-κB pathways as revealed in the in vitro and in vivo experiments [21].

In addition, Choi et al. [15] aimed to prove that chrysoeriol extracted from the leaves of *Digitalis purpurea* could be considered as a basic molecule to understand the novel anti-inflammatory mechanisms using LPS-stimulated Raw264.7 cells. They found that chrysoeriol significantly suppressed the production of NO in Raw264.7 macrophages and potently suppressed the LPS-induced inductions of iNOS gene using RT-PCR and Western blot assays. They also indicated that chrysoeriol blocked AP1 activation, which mainly resulted in inhibiting LPS-induced NO production by macrophages.

Moreover, the study performed by Prabhakar et al. [1] demonstrated that chrysoeriol isolated from *C. didymus* exhibited a significant anti-inflammatory effect in rat-induced paw edema. The aqueous extract (200 mg/kg) showed a notable decrease in the edema volume at 1 h and 3 h, whereas the ethanol extract exhibited a potent reduction in the edema volume only at 2 h, but the mechanism involved has not been elucidated. Similarly, chrysoeriol isolated from the silk of *Z. mays* could play crucial roles as an anti-inflammatory agent that has strong in vitro actions on the expressions of COX-2 proteins and iNOS in LPS-induced HaCaT human keratinocyte cells as revealed by using Western blotting [18]. Moreover, in their in vivo and in vitro experimental models, Csupor et al. [73] demonstrated that chrysoeriol obtained from the chloroform extracts of *Centaurea sadleriana* exhibited significant effects on COX-1 and COX-2 enzymes. This flavonoid isolated from the ethyl acetate fraction of the stem bark of *Passiflora foetida* *L.* might represent a novel approach to evaluate the anti-inflammatory effects, and it showed potent activity by inhibiting NO production in the macrophage cell line RAW264.7 with an IC_50_ value of 3.1 Μm [19].

In agreement with these investigations, chrysoeriol isolated from the aerial parts of *Artemisia copa* Phil. was found to limit strongly the production of inflammatory mediators in RAW 264.7 induced by LPS at 10 μM including PGE_2_ (8.2 ± 0.9 ng/mL), nitrite (397.7 ± 16 ng/mL), synovial phospholipase A_2_ (2127.9 ± 64.5 ng/mL), and COX-1 (19.3 ± 0.7 ng/mL) [16].

On the other hand, chrysoeriol isolated from the pulp of açaí fruit was found to weakly inhibit the expression of proinflammatory mediators IL-6 and TNF-α at a higher concentration (20 μM) compared to velutin (IC_50_ = 2.0 μM), which was effective in low micromole values as a strong anti-inflammatory flavone by blocking NF-κB activation and JNK and p38 phosphorylation in RAW 264.7 induced with LPS [17].

Interestingly, deglycosylation of chrysoeriol enhances its ability to inhibit pro-inflammatory cytokines that potently reduced TNF-α at doses as low as 10 μM and increases its ability to inhibit LPS-induced NF-κB transcriptional activity at a dose of 25 μM in LPS-stimulated RAW264.7 cells. Moreover, conversion of flavones from glycoside forms to aglycone forms has been shown to enhance absorption into the systemic circulation, as evidenced by the in vivo model analyzing the impacts of celery-enriched diets on flavone absorption in mice [74].

Both inflammation and apoptosis are involved in the process of cisplatin-induced nephrotoxicity. In their in vivo investigation, Qiu et al. [22] showed that chrysoeriol exhibits a reno-protection against cisplatin-induced acute kidney injury in the rat model through inhibition of NF-κB and activation of PI3K/AKT pathways, as evidenced by the evaluation of histopathological alterations and pro-inflammatory mediators.

Recently, Yoon and collaborators [20] have performed a study in order to underline other molecular pathways of chrysoeriol on LPS-induced inflammation in the RAW 264.7 cell line. The results showed that chrysoeriol treatment provides significant enhancement of LPS-induced COX-2 expression and PGE_2_ production by regulating NF-κB, AP-1, PI3K/Akt, and MAPKs mediated by toll-like receptor 4 (TLR4) and myeloid differentiation primary response 88 (MyD88) in RAW 264.7 cells [20].

### 3.3. Antidiabetic Effects

Diabetes mellitus, which is presently growing as the third highest killer in the world after cardiovascular problems, and diabetes are diseases that are currently very difficult for doctors to treat at the present time. For this reason, there has been a recent uptick in interest in the use of natural treatments for diabetes, especially those that are derived from plants [75]. In this context, Ramirez et al. [23] aimed to assess using in vitro models the pharmacological properties of chrysoeriol and other flavonoids isolated from *Tecoma stans* (L.) Juss. ex Kunth. in charge of blocking lipase activity (Table 4). They found that a mixture of chrysoeriol (96%) and apigenin (4%) was the more potent fraction (TsC2F6B) able to inhibit pancreatic lipase activity with a percentage of 80% when tested at a dose of 0.25 mg/mL. The results also indicated that chrysoeriol exhibited mixed and non-competitive inhibition with IC_50_ values of 158 µM [23].

The findings of the in vivo study conducted by Baskaran et al. [33] showed that chrysoeriol exhibited a potent antihyperglycemic activity in diabetic rats because oral supplementation of chrysoeriol to this group of rats was found to be significantly effective in reducing HbA1c compared to diabetic rats with normal blood glucose and insulin levels. The mechanism involved in this effect could be due to the release of bound insulin or alternatively to the insulin-like actions exerted by isoflavones and flavonoids.

*Otostegia persica* (Burm.) Boiss. is an Iranian plant widely used in folk medicine to treat diabetes. Tofighi et al. [27] aimed to investigate the pure agents and fractions of this herb to prove its antihyperglycemic property in vivo. In diabetic mice, chrysoeriol extracted from the methanolic extract fraction produced a substantial drop in blood glucose levels at dosages of 300 and 400 mg/kg. They suspected that this effect could be due to the synergistic actions of different drugs, particularly phenolic and flavonoid compounds, which were abundant in the methanolic extract fraction [27]. Similarly, water-soluble fraction from *Hyphaene thebaica* (L.) Mart. Epicarp, which includes chrysoeriol and other flavonoids, enhanced glucose and insulin sensitivity and led to a notable decrease in HbA1C levels in alloxan-induced diabetic rats [28]. Furthermore, as evidenced in the in vivo investigation carried out by Krishnan et al. [30], oral supplementation of chrysoeriol (20 mg/kg) to diabetic rats prevented body-weight loss and has been shown to reduce plasma glucose and insulin to near-normal ranges as compared to control animals, which might be because of its capacity to stimulate the remaining β-cells to produce insulin. On the other hand, Vinholes et al. [24] in their in vitro work have found that hydromethanolic extract from Spergularia rubra L. aerial parts contains several bioactive compounds including chrysoeriol derivatives (13%) such as arabinosyl chrysoeriol and glucosyl chrysoeriol. Moreover, the results indicated that this extract was more effective on the α-glucosidase inhibitory experiment (IC_50_ = 2.55 mg/mL), this activity could be due to the synergistic actions of different bioactive metabolites quantified in this medicinal plant as well as the content of C-glycosyl flavones. These findings are consistent with the results of the research produced by Nickavar and Abolhasani [26], in which they found that chrysoeriol isolated from the aerial parts of *Salvia virgata* Jacq. Exhibited strong antihyperglycemic activity. In a dose-dependent way, it was able to block the activity of the α-amylase enzyme, the percentage of inhibition was 58.98 ± 1.20 %, and the IC_50_ value was 1.27 (1.21–1.33) mM. Likewise, Rauter and colleagues [31] evidenced that chrysoeriol from *Genista tenera* showed an excellent antihyperglycemic performance when it was injected i.p. at a low dose (4 mg/kg/day) for a limited time (7 days) in diabetic groups, which was able to reduce significantly (*p < 0.01*) hyperglycemia 2 h after glucose loading, and oral glucose tolerance was markedly enhanced after treatment with this aglycone. Moreover, chrysoeriol and other flavonoids identified in the total extract of *Cyperus laevigatus* were revealed to display antidiabetic potency by reducing glucagon, NO, and glucose while at the same time raising insulin levels and enhancing paraoxonase activity (a protein speculated to play a role in diabetes mellitus) in diabetic rats treated with *C. laevigatus* extract [32]. Dipeptidyl peptidase IV inhibitors (DPP4i) have been proven to possess beneficial effects in the management of patients with type 2 diabetes mellitus [76,77]. Chrysoeriol could be considered a promising drug in treating diabetes as demonstrated in the investigation carried out by Purnomo et al. [25], in which chrysoeriol from ethanolic extract of *Urena lobata* L. leaf exhibited more potent DPP4i action than water extract, and the IC_50_ values were 1654.64 and 6489.88 μg/mL, respectively.

A recent study performed by Krishnan et al. [29] showed a significant effect of chrysoeriol against STZ-induced type 2 diabetes that regulates β-cell degradation in islets by a modulation of carbohydrate metabolic enzymes. In point of fact, the administration of chrysoeriol at a dosage of 20 mg/kg was successful in lowering Hb, HbA1C, and blood glucose levels while simultaneously increasing sensitivity to plasma insulin. In addition to that, chrysoeriol supplementation decreased the levels of some enzymes, especially fructose 1,6-bisphosphatase, glycogen phosphorylase, and glucose 6-phosphatase but also increased other types of enzymes including pyruvate kinase, glucose-6-phosphate dehydrogenase, hexokinase, and hepatic glycogen content. Further, using molecular docking assay, chrysoeriol exhibited a potent ligand-binding energy, and histopathological analysis showed the turnover of pancreatic β cells in rats receiving chrysoeriol. It is also worth mentioning that extensive protein glycation has been considered a result of the complexities of diabetes. Therefore, chrysoeriol has a considerable effect on HbA1C, bringing its level back to normal and enhancing the iron-binding protein in the process (Hb). It could be suggested from the outcome of the works conducted that chrysoeriol is an important promising bioactive compound to both manage diabetes and prevent hyperglycemia. Thus, it proved a protective role for vital organs confirmed by histopathological examinations such as the liver and kidneys, which can also be harmed by this abnormal condition.

### 3.4. Anti-Hyperlipidemic Properties

Investigations on the anti-hyperlipidemic effect of chrysoeriol are very rare and are needed to elucidate the activity of this bioactive compound on lipids and underlying the mechanisms involved. Only one work has the prospect to examine anti-hyperlipidemic effects of chrysoeriol, and Baskaran et al. [33] were looking to describe lipid-metabolizing enzymes and lipid profiles in STZ-induced diabetic rats treated orally by chrysoeriol. They reported a remarkable rise in lecithin cholesterol acyltransferase (LCAT) and lipoprotein lipase (LPL) activity and a significant reduction in HMG-CoA reductase activity as well as in the level of triglycerides, total cholesterol, lipoproteins, and free fatty acids (FFAs), as compared with control diabetic rats. This hypolipidemic benefit of chrysoeriol might be caused by enhanced insulin secretion and a lowered plasma glucose level that eventually resulted in an elevation of plasma LCAT activity.

### 3.5. Antioxidant Properties

Chrysoeriol has been investigated for its antioxidant effect by various studies [2,3,4,5,8,30,41,42,43]. Table 5 summarizes all research on chrysoeriol’s antioxidant properties, including studies on its origin, usage, and potential side effects method for its extraction, type of antioxidant assay, experimental approaches, and main results. Mishra et al. [2] investigated the antioxidant properties of chrysoeriol isolated from *C. didymus*, and the fractionation of an ethanolic extract utilizing petrol ether, diethyl ether, ethyl acetate, and n-butanol resulted when treated with methanol; the diethyl ether fraction forms chrysoeriol, while the ethyl acetate fraction when treated with methanol deposits its glycoside. Chrysoeriol has significant antioxidant action. O-glycosylation of chrysoeriol lowers its capacity to suppress lipid peroxidation and interaction with peroxyl radicals. However, the glycoside is a more effective scavenger of DPPH radicals and a stronger inhibitor of xanthine/xanthine oxidase than the chrysoeriol. Chrysoeriol glycoside interacts with DPPH radicals at millimolar concentrations; however, the chrysoeriol exhibited no response. Hydroxyl, azide, haloperoxyl radicals, and hydrated electrons were observed to react with both substances. It was shown that the two compounds had different rate constants for the oxidation of superoxide anion, although both chrysoeriol and its glycoside had the same rate constants for hydrated electron reactions at pH 7. Demirtas and colleagues [41], in order to test the antioxidant activity, used ferric thiocyanate, ferric ion (Fe^3+^), ferrous ion (Fe^2+^) metal-chelating activity, and DPPH free radical scavenging activity to extract three flavonoids from the leaves of A. vineale and extract them in water-soluble ethyl acetate and methanol. According to the findings, hydrogen atoms from other hydroxyl groups were used to neutralize radical species of isolated flavonoids, changing the stability of a flavonoid radical produced by the removal of a hydrogen atom from an adjacent one. The radical scavengers’ crude extract, separated flavonoids, and standard compounds significantly reduced the DPPH radical concentration (BHT, BHA, and a-tocopherol), in order to check out the protective effect of chrysoeriol on the response of osteoblasts to oxidative stress.

Kim et al. [5] isolated chrysoeriol from E. ciliata during in vivo trial research and studied the indicators of osteoblast activity and oxidative damage after incubation with chrysoeriol of osteoblastic MC3T3-E1 cells. They used Sirius Red-based colorimetric assay, ALP activity assay, calcium deposition assay, sandwich ELISA assay, b-glycerophosphate and 50 mg/mL ascorbic acid, protein lysis buffer (50 mM Tris, pH 7.5; 10 mM EDTA, pH 8; 1 mM PMSF), and lipid peroxidation assay. Chrysoeriol can protect osteoblasts from oxidative-stress-induced toxicity, for example, it reversed the cytotoxic impact of hydrogen peroxide by increasing collagen content, alkaline phosphatases activity, and calcium deposition in osteoblasts. Inhibition by ICI182780 shows that chrysoeriol may play a role in the action of oestrogens. Osteocalcin’s H_2_O_2_-induced decrease was restored when chrysoeriol was added. In the presence of hydrogen peroxide, MC3T3-E1 cells produced considerably less receptor activator of nuclear factor-kB ligand, interleukin-6, protein carbonyl, and malondialdehyde when treated with chrysoeriol. Meanwhile, Nascimento et al. [3] extracted chrysoeriol from fruits, peel, and seeds of C. frutescens by hexane and acetonitrile. The antioxidant activity was evaluated according to the results of the DPPH free radical scavenging assay, the ABTS radical cation assay, and the β-carotene bleaching test. Chrysoeriol was not as major of a component as capsaicin and dihydrocapsaicin; the acetonitrile extract of seeds had a greater chrysoeriol concentration (11.4 mg g^−1^) than the acetonitrile extract of peel, which at the same time shows low antioxidant activity compared to capsaicin and dihydrocapsaicin. Extracted from the leaves of other Capsicum, that is, *C. chinense*, in 95% methanol and then 50% methanol in twice extraction extract, Kim and Jin [4] evaluated the inhibitory activity of chrysoeriol, luteolin-7-O-glucopyranoside, and isorhamnetin-7-O-glucopyranoside. Chrysoeriol exhibited IC50 values of 11.6 ± 2.9 obtained using sEH assay and kinetic analysis. Moreover, chrysoeriol was discovered to non-competitively bind into the allosteric site of sE hydrolase, with Ki values of 10.5 ± 3.2. Park et al. examined the antioxidative potential of chrysoeriol against oxidative damage and its molecular processes by testing cell survival, ROS production, and Western blots in the RAW 264.7 cell line [42]. The antioxidant potential was evaluated by cell culture on Dulbecco’s modified Eagle medium (DMEM) supplemented with FBS and glutamine, MTS assay, formazan product measure, ROS-dependent oxidation of DCFH-DA to DCF in RAW 264.7 cells line, ROS generation, NE-PER nuclear and cytoplasmic extraction, and Western blot analysis RAW 264.7 cell lines. The chrysoeriol used was purchased. Results show that chrysoeriol strengthened the HO-1-mediated antioxidative potential through the regulation of the Nrf2/MAPK signaling pathway, and it was shown that chrysoeriol scavenged lipopolysaccharide-induced intracellular ROS generation via the activation of the extracellular signal-regulated kinase, c-Jun NH2-terminal kinases, and p38 phosphorylation. A phase II antioxidative enzyme, HO-1, was activated by chrysoeriol, which coincided with the nuclear translocation of nuclear factor-erythroid 2 p45-related factor 2. Krishnan et al. [30] evaluated the potential of chrysoeriol isolated from the leaves of *C. halicacabum*. This potential was evaluated using the glucose oxidase method, plasma insulin assay, and LPO in liver, kidney, and heart estimation. A combination of chrysoeriol and other nonenzymatic antioxidatives, such as vitamin C and vitamin E and reduced glutethione, lowered the antioxidant enzyme activity of superoxide dismutase, catalase, and glutathione peroxidase in diabetic rats. Liu et al. [8] isolated flavonoids from *E. songoricum* in ethanol extract, and their antioxidation actions were examined and used on human peripheral blood mononuclear cells to evaluate their protection against DNA damage and inhibition of lipid peroxidation PBMC separation by lymphocyte separation medium. Comet assay, lipid peroxidation induced by Fe^2+^/ascorbate in rat liver, microsoma in rats’ liver, and single-cell gel electrophoresis to detect damaged cells’ lipid peroxidation product thiobarbituric acid reactive substances (TBARS) was determined. The findings indicated that the majority of flavonoids have concentration-dependent antioxidative actions, and flavonoids play a role in preventing DNA damage and lipid peroxidation from oxidative assault. Chrysoeriol has a value of IC_50_ = 11.9 µM. Singh and Gupta [43] extracted chrysoeriol from *Abutilon indicum* L. in ethanol extract to evaluate its antioxidative status and lipid peroxidation of its flavonoids. Results show that with chrysoeriol and chrysoeriol-7-O-beta glucopyranoside, among other components, peroxidative damage was minimal in rats’ liver, and there was an observed decrease in liver and blood levels of enzymic and non-enzymic antioxidants, such as superoxide dismutase, catalase, glutathione peroxidase, glutathione reductase, glutathione-S-transferase, glutathione-one, vitamin C, vitamin E, ceruloplasmin, β-carotene, and other antioxidants.

### 3.6. Antimicrobial Activities

#### 3.6.1. Antibacterial Activity

Various studies showed the antibacterial viability of chrysoeriol from methanol, hexane, and acetonitrile extracts from *C. frutescens, Cynara syriaca* L., and *Salvia palaestina* Benth. and from biotransformation as well [3,34,35]. An overview of all research that looked into chrysoeriol’s antibacterial activity, including its source, kind of antibacterial assay, strains examined, and major findings, may be seen in the table below. Chrysoeriol’s antibacterial ability against Gram-positive and Gram-negative bacteria has been studied by a number of writers (Table 6). According to the literature, Bashyal et al. [34] used a biotransformed chrysoeriol in Gram-positive strains of *Staphylococcus aureus, Bacillus subtilis, Enterococcus faecalis,* and *Kocuria rhizophilla* and Gram-negative strains of *Salmonella enterica, Klebsiella pneumonia, Escherichia coli, and Proteus hauseri.* Chrysoeriol manifested antimicrobial activity in a disc diffusion assay at the concentration of 40 µg per disc. Authors found MIC values of 20 µg/mL for luteolin and 1.25 µg/mL for chrysoeriol against Staphylococcus aureus, while the values against Proteus hauseri were 50 µg/mL for luteolin and 30 µg/mL for chrysoeriol. However, Nascimento et al. [3] extracted chrysoeriol from fruits, peel, and seeds of *C. frutescens* by hexane and acetonitrile and then used it in broth micro-dilution assay on *Enterococcus faecalis, Bacillus subtillis,* and *Staphylococcus aureus.* Chrysoeriol was of significative activity as showed by MIC values: *Escherichia coli* MIC = 0.06 µg/mL; *Pseudomonas aeruginosa* MIC = 0.12 µg/mL; *Klebsiella pneumonia* MIC = 0.25 µg/mL; *Enterococcus faecalis* MIC = 1µg/mL; *Bacillus subtillis* MIC = 1 µg/mL; *Staphylococcus aureus* MIC = 0.25 µg/mL. Karasin et al. [30] tested chrysoeriol from leaves and flowers of *C. syriaca* extracted with methanol using disc diffusion method on strains of *Streptococcus pyogenes, Staphylococcus aureus, Pseudomonas aeruginosa,* and *Escherichia coli.* Chrysoeriol among other flavonoids showed an inhibition zone <12 mm. Miski et al. [35] determined the antibacterial activity of flavonoids (salvigenin, eupatilin, apigenin 7,4′-dimethyl ether, luteolin, genkwanin,6,7,3′,4′-tetramethoxyflavone, cirsimaritin, chrysoeriol, apigenin, luteolin, apigenin 7-glucoside, apigenin 7-glucuronide, luteolin 7-glucoside, luteolin 7-glucuronide, chrysoeriol 7-glucoside, and chrysoeriol7-glucuronide) by disc diffusion method on *Staphylococcus* *aureus, Staphylococcus* *epidermitis, Escherichia coli, Klebsiella pneumoniae, Proteus vulgaris,* and *Pseudomonas aeruginosa.* Chrysoeriol and flavonoids were extracted from *S. palaestina* leaves on alcohol extract. Among them, cirsimaritin showed a high activity against *Staphylococcus* *aureus, Staphylococcus epidermidis, Escherichia coli, Klebsiella pneumoniae, Proteus vulgaris,* and *Pseudomonas aeruginosa,* while the others have little or no activity against the same bacterial strains.

#### 3.6.2. Antifungal Activity

Jang et al. [78] chose the cultivars with the highest extraction efficiency after having their chrysoeriol from *Oryza sativa L*. (rice) infected with the white-backed planthopper and then being tested with MeOH. The fungi strains *Cladosporium herbarum, Alternaria tenuissima, Cladosporium cladosporioides, Curvularia lunata,* *Fusarium oniliforme, Alternaria padwickii, Gibberella zeae, Fusarium graminearum, Pythium graminicola, Pythium ultimum,* and *Rhizoctonia cerealis* were tested by microdilution method. Chrysoeriol had an antifungal activity against *Fusarium graminearum* and *Pythium graminicola.* At first week after inoculation, *Fusarium graminearum* had an inhibition rate of 10.9% when treated with 100 ppm of chrysoeriol, and when treated with 500 ppm the inhibition was 26.6%, while the value was 28.7% when treated with 1000 ppm. Whilst the inhibition rates of *Pythium graminicola* were 22.6% at 100 ppm of chrysoeriol, at 500 ppm the inhibition was 48.54%, while the value was 62.27% when treated with 1000 ppm after 1 week of inoculation. However, after 2 weeks of inoculation, the inhibition rate was low to values of 0.2%, 1.9%, and 5.1%. Chrysoberyl decreased the growth of tested fungi strains.

### 3.7. Chrysoeriol Effect on Prevention and Treatment of Vascular Diseases

Cha et al. [7] examined how chrysoeriol affected the number of human aortic smooth muscle cells that were produced in culture (HASMC). It strongly inhibited platelet-derived growth factor (PDGF) at a concentration of 20 ng/mL, which triggered migration and [3H]-thymidine combination into DNA at doses of 5 and 10 μM that were beyond the range of cytotoxicity. Chrysoeriol was able to suppress phosphorylation of PDGF beta-receptors in a way that was concentration dependent. Additionally, it was able to prevent PDGF from stimulating the dissociation of actin filaments. Therefore, in the same manner, the downstream signal transduction pathways of PDGF-(Rβ), including ERK1/2, p38, and Akt phosphorylation, were likewise blocked by chrysoeriol.

Chrysoeriol was expected as a novel cardio protective agent against doxorubicin-induced cardiotoxicity. An in vitro assay was carried out by Liu et al. [38] to assess the protective effect of chrysoeriol extracted from the leaves and caudexes of *E. songoricum* anti-apoptosis, and anti-cell death against doxorubicin-induced H9c2 cell death lactate dehydrogenase release was measured using an MTT test with Hoechst 3325 8 staining. At a dosage of 20 µg/mL, chrysoeriol dramatically reduced doxorubicin-induced apoptosis and cell death, lowered MDA content in the supernatant of doxorubicin-treated H9 c2 cells, and enhanced SOD (SOD-GPx) and GPx (GPx-SOD) activity to normal levels.

The bronchodilator effect of chrysoeriol was tested by Khan and Gilani [39] from *A. linearis* (rooibos teas) on an aqueous extract. After adding 1.5 mL of the extract solution to an equivalent amount of a solution of 2% AlCl3 ’ 6H2O in methanol, the resulting mixture was evaluated in vitro on isolated tissue preparations that were placed in the appropriate physiological salt solutions and aerated with carbogen. In order to conduct in vivo investigations, rats were given pentothal sodium to put them to sleep, and then their blood pressure was recorded by cannulating their carotid arteries. Results show that among the tested pure compounds of rooibos, chrysoeriol (luteolin 3′-methyl ether) showed a selective bronchodilator effect, mediated predominantly through KATP channel activation along with weak Ca++ antagonist mechanisms.

### 3.8. Anti-Osteoporosis Activity of Chrysoeriol

According to the findings of Tai et al., chrysoeriol has a significant favorable impact on the augmentation of osteoblast activity, which might be effective in the prevention of osteoporosis. In order to investigate the effects of chrysoeriol’s biological activities on the osteoblastic MC3T3-E1 cells, chrysoeriol was extracted from a methanol extract of the dried leaves of *E. ciliata*. Chrysoeriol was found to be the primary ingredient in this extract. Chrysoeriol induced a considerable elevation in the activity of alkaline phosphatase, as well as an increase in collagen content and nodule mineralization. This resulted in a large rise in the proliferation of MC3T3-E1 cells [6].

### 3.9. Neuroprotective Activity

Shao et al. [36] verified the role of chrysoeriol on neuroprotection. It took three days for the rats to undergo temporary mid-brain artery blockage surgery and chrysoeriol treatment (3 mg/kg, 6 mg/kg, and 9 mg/kg) administration. The effects on the Wnt/β-catenin pathway were evaluated along with the following measures: neurological deficit scores (1 day before, 2 h after, and 6 h after the last chrysoeriol administration) on middle cerebral artery occlusion, hematoxylin and eosin staining, triphenyl tetrazolium chloride staining, an enzyme-linked immune sorbent assay (ELISA), gaspase-3 immunofluorescence, terminal deoxynucleotidyl transferase-mediated dUTP nick-end labeling assay, Nissl staining, Western blotting and an immunofluorescence assay, and Wnt inhibitor. According to the findings of the clinical trials, chrysoeriol helped rats recover from neurological deficits, alleviated neurological damage, reduced the area of ischemia, inhibited excessive pro-inflammatory cytokine production (tumor necrosis factor, interleukin (IL) 1 β and IL 6), and regulated oxidative stress (malondialdehyde, superoxide dismutase, and glutathione). Moreover, the Wnt inhibitor known as Dickkopf-related protein 1 proved that the neuroprotective effects of chrysoeriol are a direct result of the Wnt/β-catenin signaling pathway.

### 3.10. Chrysoeriol Effect on the Cellular Models of Parkinson’s Disease

After observing the chrysoeriol’s neuroprotective impact against MPP+ treatment brought about by the activation of the PI3K/Akt pathway, the chrysoeriol was evaluated for its potential use as a therapeutic agent in the treatment of Parkinson’s disease. It has come to light that chrysoeriol inhibits the apoptosis caused by MPP+ in SH-SY5Y cells. As a consequence, MPP+ causes a reduction in the phosphorylation of Akt (Ser473) and mTORC1 (Ser2448), but this effect may be counteracted by chrysoeriol. Additionally, chrysoeriol maintains the mitochondrial membrane potential (MMP) while protecting mitochondrial localization from the effects of MPP+ [37].

### 3.11. Anti-Insecticidal Activity

#### 3.11.1. Anti Pea Alphid *A. pisum* Activity

Chrysoeriol glycosides and other flavonoids were discovered in the aerial portions of alfalfa (*M. sativa*) (*Fabaceae*) Radius cv, both in areas that were not affected by the pea aphid *A. pisum* Harris and in areas that were infested by the pea aphid. *M. sativa* aerial parts that had not been infected by *A. pisum* or that had been infested by *A. pisum* were collected, freeze-dried, powdered, and then stored in a desiccator in the dark until they were tested. Chrysoeriol glycosides with other flavonoids were obtained using methanol and MeOH extracts. Golawska et al. [52] had shown that chrysoeriol glycosides affected pea aphid daily fecundity per female, and the level of chrysoeriol glycosides affected ingestion of xylem sap by the aphid.

#### 3.11.2. Nodulation Genes Transcription Induction

The aqueous rinses of Moapa 69 *M. sativa* seeds were collected and tested in an experiment conducted by Hartwig et al. [9] to determine whether or not they might induce a nodABC-IacZ fusion in *R. meliloti*. The entire nodulation gene-inducing activity that was released from seeds at rates that were 100 times greater than those that were released from the roots of 72 h old seedlings occurred within the first four hours of imbibition. Chrysoeriol and other flavonoids were isolated and identified by spectroscopic investigations. After being compared with legitimate standards, it was determined that the flavonoids found in seed rinses were active nodulation gene-inducing flavonoids. *R. meliloti* experienced a half-maximal induction of nodABC-IacZ at a concentration of chrysoeriol equal to five nanomolar. According to the findings, the majority of the activity responsible for activating nodulation genes is linked to the breakdown of chrysoeriol. The fact that they are present in high amounts lends credence to the hypothesis that they play a substantial role in the nodulation gene-inducing activity that occurs in the soil.

#### 3.11.3. Anti *S. litura* Activity

The insecticidal potential and mechanism of action of chrysoeriol, which was isolated from *M. suavis*, were examined against *S. litura* (Fabricius) by Ruttanaphan et al. [40]. *M. suavis* leaves and twigs were sun-dried, and chrysoeriol was extracted using dichloromethane and EtOH–acetone. Early second instar larvae were treated with 1 μL of chrysoeriol in the dorsal thoracic area. The treatment group included contained crude extract dilutions. The acetone-only group was the negative control, while the cypermethrin-only group was the positive control. To test for enzyme activity, surviving S. litura second instar larvae were treated with chrysoeriol at LD30 for 24 or 48 h before being used for enzyme preparation, enzyme activities related to detoxification or neurological functions, and the Bio-Rad protein assay. Glutathione S-transferase activity was determined using crude enzymes in PPB, and acetylcholinesterase activity was tested using PPB enzyme crudes. Chrysoeriol is poisonous to S. litura larvae in their second instar, with LD_50_ values of 6.99 and 6.51 µg/larva recorded during 24 and 48 h periods, respectively. Carboxylesterases and glutathione S-transferase, as well as acetylcholinesterase, were shown to be strongly inhibited by chrysoeriol in mode-of-action assays.

#### 3.11.4. In Vivo Pharmacokinetic Investigations

It has been reported by Chen and colleagues [79] that they developed an HPLC-UV method for simultaneous determination of rat plasma chrysoeriol concentration after orally administering the synthesized compound to rats, and the results show that with co-administration of cathodol-O-methyltransferase, the concentration of chrysoeriol in the plasma was significantly reduced. This was accomplished by using an HPLC system equipped with an online degasser, an auto-sampler, a VWD detector, and a thermostatic column compartment.

In molecular dynamic modeling and ADMET prediction, Liu et al. [44] have shown that chrysoeriol has excellent stability and pharmacokinetic behavior. ITC, multi-spectroscopic techniques, molecular simulations, and ADME were all utilized to study the interaction between the chrysoeriol and the enzyme, xanthine oxidase, in the presence of DMSO or ethanol extracts of chrysoeriol purchased from suppliers. Research shows chrysoeriol served as an immediate and competitive inhibitor of the enzyme, driven by hydrogen bonding and hydrophobic interactions with the xanthine oxidase substrate. Static quenching of xanthine oxidase resulted in a substantial fluorescence quenching impact and a conformational change in the enzyme. There were interactions with amino acid residues that are critical for binding to XO, and this resulted in an increased affinity for XO, as shown in the docking studies.

## 4. Conclusions and Perspectives

Chrysoeriol’s key pharmacological properties have been documented and emphasized in this article. This natural molecule’s outstanding biological effects, particularly against tumor cell lines, have been established in several published studies. Traditional medicine has utilized this herb extensively to cure diabetes. Several works of pharmacological research have shown the validity of this ancient wisdom. Chrysoeriol blocks and inhibits important pathways of colon cancer, gastric cancer, human lung cancer, pancreatic cancer, C6 glioma cells, and breast cancer with diverse target locations, according to molecular and cellular investigations. Anticancer drugs might benefit from its inclusion. Molecularly, this compound has the potential to be a beneficial bioactive component in the treatment of cancer and neurological illnesses due to its antibacterial, anti-inflammatory, antioxidant, and anti-hyperlipidemic capabilities. Its pharmacokinetic and pharmacodynamic qualities need to be studied further before it can be used as a chemotherapy medication. In addition, more toxicological studies are needed to confirm its safety.

## Figures and Tables

**Table 1:** Sources of chrysoeriol.

Source	Country	Part	Extract	Extraction Methods	References
*Melientha suavis* Pierre	Thailand	Leaves and twigs	Dichloromethane	Maceration	[40]
*Olea europaea* L.	Indonesia	Leaves	Ethyl acetate	Maceration	[45]
*Capsicum chinense* Jacq.	Korea	Leaves	Methanol	Maceration	[4]
*Narthecium ossifragum* (L.) Huds.	Norway	Flowers	Methanol	Maceration	[46]
*Glandularia selloi* (Spreng.) Tronc.	Brazil	Aerial parts and roots	Methanol	Maceration	[47]
*Phoenix dactylifera* L.	Morocco	Fruit flesh and pits	Hexane and methanol	Soxhlet apparatus	[48]
*Graptophyllum grandulosum* Turill	Cameroon	Aerial parts	Methanol	Maceration	[49]
*Cardiospermum halicacabum* L.	India	Leaves	-	-	[33]
*Arum palaestinum* Boiss.	Jordan	Aerial parts	Diethyl ether	Maceration	[50]
*Artemisia arborescens* L.	Algeria	Aerial parts	*n*-butanol	Maceration	[51]
*Capsicum frutescens* L.	Brazil	Seeds, peel, and whole fruits	Hexane and acetonitrile	Ultrasonication	[3]
*Allium vineale* L.	Turkey	Leaves	Methanol and ethyl acetate	Decoction	[41]
*Medicago sativa* L.	Poland	Aerial parts	-	ASE 200 Accelerated Solvent Extractor	[52]
*Medicago sativa* L.	Poland	Aerial parts	-	ASE 200 Accelerated Solvent Extractor	[53]
*Saussurea alpina* L.	Russia and Mongolia	Aerial parts	Methanol	Maceration	[54]
*Saussurea daurica* L.					
*Saussurea laciniata* L.					
*Saussurea pricei* L.					
*Saussurea pseudo-alpina* L.					
*Saussurea salicifolia* L.					
*Saussurea salsa* L.					
*Myoporum bontiodes* spp.	Japan	Flowers and leaves	Methanol	Maceration	[55]
*Eurya cilliata* Merr.	Vietnam	Leaves	-	Maceration	[5]
*Eurya ciliata* Merr.	Vietnam	Leaves	Methanol	Maceration	[38]
*Eremosparton Songoricum* (Litv) Vass.	China	Leaves and caudexes	Methanol and ethyl acetate	Maceration	[38]
*Phlomis caucasica* Rech.	Iran	Aerial parts	Methanol	Soxhlet apparatus	[56]
*Zea mays* L.	Japan	Styles	Methanol and aqueous extracts	Maceration	[57]
*Phlomis fruticosa* L.	Balkans	Leaves	Methanol	Decoction	[58]
*Schouwia thebica* Webb	Egypt	Aerial parts	Ethanol	Maceration	[59]
*Alhagi maurorum Medik.*	Egypt	Aerial parts	Ether, chloroform, ethyl acetate, and *n*-butanol	Maceration	[59]
*Aspalathus linearis* (Burm.f.) R.Dahlgr.	South Africa	Leaves	aqueous extracts	Decoction	[39]
*Cynara syriaca* L.	Turkey	Leaves	Ethyl acetate	Soxhlet apparatus	[60]
*Phlomis olivieri* Benth.	Iran	Aerial parts	Ethyl acetate and *n*-butanol	Percolator	[61]
*Phlomis persica* Boiss.					
*Sideritis ozturkii* Aytac & Aksoy	Turkey	Aerial parts	Acetone	Maceration	[62]
*Salix matsudana* spp.	China	Leaves	Ethanol	Maceration	[2]
*Coronopus didymus* (L.) Sm.	India	Whole plant	Petrol ether, diethyl ether, ethyl acetate, and *n*-butanol	Soxhlet apparatus	[2]
*Coronopus didymus* (L.) Sm.	India	Whole plant	Petrol ether, diethyl ether, ethyl acetate, and *n*-butanol	Soxhlet apparatus	[1]
*Medicago sativa* L.	Poland	Aerial parts	Methanol	Maceration	[63]
*Phlomis nissolii* L.	Turkey	Aerial parts	Methanol	Maceration	[64]

**Table 2:** Anticancer effects of chrysoeriol.

Cell Lines	Key Results	References
Cell line A549 from lung cancer	IC_50_ = 16.95 Μm. Inhibited growth of A549 cells. Induced autophagy. Caused sub-G_1_/G_0_ arrest cells. Decreased cell migration and invasion. Inhibited tumor growth in vivo at the dosage of 50 mg/Kg. Inhibited the expression of p-p38 and p-ERK1/2 pathway.	[11]
Colon cancer cells HT-29, uterine cancer cells HeLa, and lymphoma cells HL-60	Selectively killed leukemic cells. Up-regulated NFAT transcriptional pathways. Amplificated intracellular ROS in HL-60 cells. Exhibited cytotoxic potential and NF-κB p65 inhibition in HL-60 cells.	[5]
HeLa-UGT1A9 cells A549 and HepG2 cells Human-expressed UGT enzymes	Ko143 inhibited the efflux of glucuronides. Ko143 increased intracellular glucuronides. BCRP-mediated chrysoeriol glucuronide. Chrysoeriol with Ko143 decreased the A549 and HepG2 cell viability.	[10]
Human gastric cancer AGS cells	Exhibited a level of cytotoxicity against AGS cells that was moderate. Cleavage of poly (ADP-ribose) polymerase that is induced by an external stimulus (PARP). Caspase-3 and caspase-8 that have been activated.	[65]
SW1990 pancreatic cancer cells	IC_50_ = 56.35 ± 6.96 μM. Exhibited stronger pro-apoptosis effect against SW1990 cells. Targeted potentially to BCL-2. Identified as a BH3 mimetic.	[66]
C6 glioma cells	Decreased cell viability. Induced apoptosis. Bax/Bcl-2 ratio increased. Caspases-3/caspase-3 ratio cleaved. Reduced the phosphorylation of PI3K, Akt, and mTOR expression.	[13]
Renal carcinomas A-498 and 769-P, as well as colon cancers Caco-2	Exhibited anti-proliferative effect. Inhibited cell growth of A-498 (91%) and 769-P cells (84%). Repressed cell growth of Caco-2 colon cancer cells (IC_50_ = 239.7 ± 43.2 μg/mL).	[12]
In silico against several cancer receptors	IC_50_ = 8.26 µg/Ml. P-Glycoprotein-1, cyclin-dependent kinase-2, and phosphoinositide-3-kinase receptors showed anticancer action. Induced antiproliferative activity for the Cyclooxygenase-2/phosphoenolpyruvate carboxykinase receptors.	[14]
Melanogenesis in B16F10 cells	Increased the expression of TRY/TRP-1/TRP-2. Up-regulated the expression of MITF. Suppressed the phosphorylation of ERK/AKT. Increased the phosphorylation of p38 MAPK/GSK-3β/β-catenin/PKA. Decreased the production of β-catenin.	[67]
MCF-7 breast cancer cells	Significantly inhibited TNFα-induced EGR-1 expression. Significantly inhibited TNFα-induced CYP19 expression. Suppressed ERK1/2-mediated EGR-1 expression.	[68]
HeLa cells	Inhibited the 9-cis-RA induced RXRα transcription. Exhibited concentration-dependent inhibitory effects (12.5–50 μmol/L).	[69]

**Table 3:** Anti-inflammatory effects.

Experimental Approaches	Key Results	References
TPA (12-O-tetradecanoylphorbol-13-acetate)—a mouse model of induced otitis media	Skin irritation was reduced to a more manageable level. Thinning of the earlobes. Reduced ear heaviness. Inflamed ear tissues had a lower number of inflammatory cells. Reduced protein levels of Ser536, Tyr705, iNOS, COX-2, IL-6, IL-1β, and TNF-α.	[21]
RAW264.7 cells activated with LPS	The synthesis of NO and prostaglandin E_2_ was reduced due to lower amounts of Ser536, Tyr705, iNOS, COX-2, IL-6, IL-1β, and TNF-β in the proteins. Inhibited the phosphorylation of inhibitor of κB (Ser32)/ p65 (Ser536)/Janus kinase 2 (Tyr1007/1008). Decreased nuclear localization of p50/p65/STAT3. Down-regulated mRNA levels of pro-inflammatory cytokines IL-6/IL-1β/ TNF-α.	
LPS-stimulated Raw264.7 cells	The cells treated with it were unable to release NO. Inhibited the LPS-induced inductions of iNOS gene. Suppressed AP-1 activation.	[15]
Carrageenan-induced hind paw edema model	Significantly reduced the edema volume at 2 h (ethanol extract). Significantly reduced the edema volume at 1 h and 3 h (aqueous extract).	[1]
LPS-stimulated RAW264.7 cells	Inhibited NO production (397.7 ± 16 ng/mL). Inhibited COX-1 activity (19.3 ± 0.7 ng/mL). Partially inhibited sPLA2 (2127.9 ± 64.5 ng/mL). Partially inhibited synovial phospholipase A_2_ activity.	[16]
RAW264.7 cells activated with LPS	IL-6 and TNF-α expression was only moderately reduced at 20 μM. Only slightly inhibited p38 phosphorylation. No inhibitory effect against JNK phosphorylation.	[17]
LPS-induced HaCaT human keratinocyte cells	Strongly inhibited LPS-induced iNOS and COX-2.	[18]
LPS-stimulated RAW264.7 cells	IC_50_ = 3.1 μM. Significantly inhibited NO production.	[19]
Induced Acute Kidney Injury in A Rat Model	Exhibited a reno-protection against cisplatin-induced acute kidney injury. Inactivated NF-κB pathway. Activated PI3K/AKT pathway.	[22]
RAW 264.7 cell line	Significantly inhibited LPS-induced PGE2 and COX-2. Activated transcription factors NF-κB and AP-1. Abolished LPS-induced phosphorylation levels of PI3K/Akt/MAPK. Inhibited the LPS-induced activation of TLR4/MyD88.	[20]

**Table 4:** Antidiabetic effects.

Experimental Approach	Key Results	References
Spectrophotometric analysis	IC_50_ = 158 µM. Inhibited pancreatic lipase activity. Exhibited mixed and non-competitive inhibition.	[23]
Streptozotocin (STZ)-induced diabetic rats	Showed an antidiabetic effect. Significantly decreased the levels of glucose. HbA1c levels were significantly reduced. Insulin levels were significantly raised.	[33]
Streptozotocin-induced diabetic mice	Exhibited antihyperglycemic effect. At dosages of 300 and 400 mg/kg, this drug reduces blood glucose levels.	[27]
Gly-pro-p-nitroanilide and vildagliptin	Inhibited dipeptidyl peptidase IV activity.	[25]
Streptozotocin (STZ)-induced diabetic rats	Reduced plasma glucose level. Reduced Hb and HbA1C level. Increased insulin sensitivity in the bloodstream. Glucose 6-phosphatase, fructose 1,6-bisphosphatase, and glycogen phosphorylase are among the enzymes that have been down-regulated. Hexokinase, glucose-6-phosphate dehydrogenase, pyruvate kinase, and hepatic glycogen levels were among the enzymes that were shown to have increased activity. Showed greatest ligand binding energy. Up-regulated the carbohydrate metabolic enzymes. Showed the turnover of pancreatic β cells.	[29]
α-Amylase inhibition test	IC_50_ = 1.27 (1.21–1.33) Mm. % of inhibition = 58.98 ± 1.20. Inhibited the α-amylase activity concentration dependently.	[26]
α-Glucosidase inhibitory assay	IC_50_ = 2.55 mg/mL. Showed α-glucosidase inhibition.	[24]
Rats were made diabetic with the drug streptozotocin (STZ).	Exhibited antidiabetic effects in rats that had STZ diabetes produced in them. Lowered concentrations of glucose, glucagon, and NO in the serum. An elevated insulin concentration prompted an increase in paraoxonase activity.	[32]
Streptozotocin (STZ) caused diabetic rats Oral glucose tolerance test	Significantly lowered the blood glucose levels (4 mg/kg b.w./day). Exerted an efficient protection on liver and kidney. Significantly enhanced oral glucose tolerance.	[31]

**Table 5:** Antioxidant activities of chrysoeriol.

Molecule	Origin	Used Methods	Experimental Approaches	Key Results	References
Chrysoeriol Chrysoeriol-6-O-acetyl-40-b-d-glucoside	*Coronopus didymus*	Ethanol extract Diethyl ether extract Ethyl acetate n-butanol extract Methanol	DPPH. Lipid peroxidation brought about by exposure to g-radiation, iron (III), and iron (II).	Inhibit enzymatically produced superoxide anion by xanthine/xanthine oxidase system. Antioxidant activity is high in chrysoeriol. Inhibition of lipid peroxidation and peroxyl radical reactions are reduced by O-glycosylation of chrysoeriol. The glycoside is more effective than the aglycone at scavenging DPPH radicals and inhibiting xanthine/xanthine oxidase.	[2]
Chrysoeriol-7-O-[2′’-O-E-feruloyl]-b-D-glucoside chrysoeriol	*Allium vineale* L. (Leaves)	Water-soluble ethyl acetate Methanol in hexane (0–100% ethyl acetate and methanol)	The ferric thiocyanate method, ferric ion (Fe^3+^)-reducing antioxidant power assay (FRAP). Ferrous ion (Fe^2+^) metal chelating activity, DPPH.	Free hydroxyl groups were a source of hydrogen atom(s) in the neutralization of radical species of isolated flavonoids, affecting the stability of a flavonoid radical generated by the abstraction of a hydrogen atom from another hydroxyl group. The ability of crude extract, separated flavonoids, and standard compounds to scavenge free radicals resulted in a statistically significant (*p* < 0.01) reduction in the concentration of DPPH radical (BHT, BHA, and a-tocopherol).	[41]
Chrysoeriol	*Eurya cilliata*		Sirius Red-based colorimetric assay. ALP activity assay. Calcium deposition assay. Sandwich ELISA assay. B-glycerophosphate and 50 mg/ ml ascorbic acid. Buffer for the lysis of protein (50 mM Tris, pH 7.5; 10 mM EDTA, pH 8; 1 mM PMSF). Lipid peroxidation assay.	Chrysoeriol can protect osteoblasts from oxidative stress-induced toxicity. In the presence of H_2_O_2_, the cytotoxic impact that was caused by H_2_O_2_ was neutralized by chrysoeriol, which also led to an increase in the osteoblasts’ collagen content, alkaline phosphatase activity, and calcium deposition. The fact that the impact of chrysoeriol may be blocked by ICI182780 shows that it may be somewhat involved in the action of estrogen. H_2_O_2_-induced reduction of osteocalcin was recovered in the presence of chrysoeriol. In the presence of H_2_O_2_, the generation of receptor activator of nuclear factor-kB ligand, interleukin-6, protein carbonyl, and malondialdehyde was considerably reduced by chrysoeriol in MC3T3-E1 cells.	[5]
Chrysoeriol	*Capsicum frutescens*	Hexane extract Acetonitrile extract	Assay for the Removal of Free Radicals Using the DPPH. Radical Cation Assay with ABTS. β-Carotene Bleaching Test.	The acetonitrile extract of the seeds, skin, and entire fruits contained capsaicin as the primary component, with dihydrocapsaicin and chrysoeriol also present in abundant amounts. The acetonitrile extract of seeds had a greater concentration of chrysoeriol (11.4 mg g^−1^) than the acetonitrile extract of peel. However, chrysoeriol exhibited a lower level of antioxidant activity in comparison to capsaicin and dihydrocapsaicin.	[3]
Chrysoeriol	*Capsicum chinense* (leaves)	Methanol extract Twice extraction	Epoxide hydrolase sEH assay.	IC_50_ values of 11.6 ± 2.9. Chrysoeriol was discovered to bind into the allosteric site of sE hydrolase in a non-competitive manner, with Ki values ranging from 10.5 ± 3.2.	[4]
Chrysoeriol	Purchased		Dulbecco’s modified Eagle medium (DMEM) containing fetal bovine serum (FBS) and glutamine was used for the cell culture. MTS assay Formazan product measure ROS-dependent oxidation of DCFH-DA to DCF in RAW 264.7 cells line. ROS generation. NE-PER Nuclear and Cytoplasmic Extraction. Western blot analysis RAW 264.7 cells line.	Through the modulation of the Nrf2/MAPK signaling pathway, chrysoeriol was able to improve the antioxidative potential that is mediated by HO-1. HO-1 overexpression caused by chrysoeriol, which was mediated via extracellular signal-regulated kinase, c-Jun NH2-terminal kinase, and phosphorylation of p38. Chrysoeriol. Scavenged intracellular ROS generation caused by lipopolysaccharide in a dose-dependent manner, without causing any cytotoxicity. Induction of nuclear factor-erythroid 2 p45-related factor 2 into the nucleus by chrysoeriol resulted in the production of the phase II enzyme known as heme oxygenase-1, which is responsible for antioxidative activity. Chrysoeriol also promoted the production of heme oxygenase-1.	[42]
Chrysoeriol	*Cardiospermum halicacabum* leaves		Streptozotocin (STZ)-treated diabetic rats. Glucose oxidase method. Plasma insulin assay. Biochemical measurements. LPO in liver, kidney, and heart estimation.	In diabetic rats, the levels of non-enzymatic antioxidants such as vitamin C, vitamin E, and reduced glutathione, as well as the activities of the enzymatic antioxidants superoxide dismutase, catalase, and glutathione peroxidase, were reduced.	[30]
Chrysoeriol	*Eremosparton songoricum*	Ethanol extract	PBMCs separation by lymphocyte separation medium. Comet assay. Lipid peroxidation induced by Fe^2+^/ascorbate in rat liver. Microsoma in rats’ liver. Single-cell gel electrophoresis to detect damaged cells. Thiobarbituric acid reactive substances (TBARS) were shown to be a lipid peroxidation product.	Oxidative activity on the protection of DNA damage and lipid peroxidation. IC_50_ = 11.9 µM.	[8]
Chrysoeriol Chrysoeriol-7-O-beta glucopyranoside	*Abutilon indicum* L.	Leaves shade dried and pulverized Ethanol (95% *v*/*v*) extract NMR and mass spectrometric evaluation	Up to 3g/kg of compound administration to rats in graded doses. By performing a heart puncture, blood samples were taken, and the serum was analyzed for a number of different antioxidant indicators. Lipid peroxidation monitoring in both serum and liver to evaluate unsaturated fatty acid formation in the hepatic cells.	Chrysoeriol and Chrysoeriol-7-O-beta glucopyranoside among other components show that peroxidative damage was minimal in both liver and serum decrease in liver and blood serum in the levels of enzymic and non-enzymic antioxidants such as superoxide dismutase, catalase, glutathione peroxidase, glutathione reductase, glutathione-S-transferase, glutathione, vitamin C, vitamin E, ceruloplasmin, and β-carotene, which are all important factors.	[43]

**Table 6:** Antibacterial activities of chrysoeriol.

Origin	Used Methods	Tested Strains	Key Results	References
*Escherichia coli* cells by microbial biotransformation	Disc diffusion assay 40 µg per disc	Gram positive *Staphylococcus* *aureus* *Bacillus subtilis* *Enterococcus faecalis* *Kocuria rhizophilla* Gram negative *Salmonella enterica* *Klebsiella pneumonia* *Escherichia coli* *Proteus hauseri*	MIC = 1.25 µg/mL against *Staphylococcus aureus.* MIC= 30 µg/mL against *Proteus hauseri.*	[34]
*Capsicum frutescens* *(Pimenta Malagueta)*	Broth micro-dilution assay	Gram negative *Escherichia coli* *Pseudomonas aeruginosa* *Klebsiella pneumonia* Gram positive *Enterococcus faecalis* *Bacillus subtillis* *Staphylococcus aureus*	MIC = 0.06 µg/mL MIC = 0.12 µg/mL MIC = 0.25 µg/mL MIC = 1 µg/mL MIC = 1 µg/mL MIC = 0.25 µg/mL	[3]
*Cynara syriaca* Boiss	Disc diffusion method	Gram positive *Streptococcus pyogenes* *Staphylococcus aureus* Gram negative *Pseudomonas aeruginosa* *Escherichia coli*	Chrysoeriol, among other flavonoids, showed an inhibitory zone <12 mm. The leaf extract showed the maximum effectiveness against *c. albicans*, with a minimum inhibitory concentration (MIC) value of 250 g mL^−1^ and an inhibition zone width of 12.5 ± 0.7 mm inhibition zone diameter and 250 µg mL^−1^ MIC value.	[30]
*Salvia palaestina*	Disc diffusion method	Gram positive *Staphylococcus aureus, Staphylococcus epidermidis* Gram negative *Escherichia coli* *Klebsiella pneumoniae* *Proteus vulgaris Pseudomonas aeruginosa*	Chrysoeriol was isolated (18 mg from 500 g of dried leaves) but not tested on the cited bacterial stains.	[35]

## Data Availability

Not applicable.
